# "The Hospital" Nursing Mirror

**Published:** 1901-02-02

**Authors:** 


### "The Hospital" Nursing Mirror.

Being the Nursing Section of "The Hospital."

[Contributions for this Section of "The Hospital" should be addressed to the Editor, "The Hospital" Nursing Mirror, 28 & 29 Southampton Street Strand, London, W.C.]

### Notes on News from the Nursing World.

### QUEEN VICTORIA'S NURSE.

It has been stated by some of the daily papers that Queen Victoria was not attended in her illness by a trained nurse. We are able to say, however, that the services of Miss Mary Ann Soal, the resident nurse on her Majesty's estate were available. Miss Soal has been at Osborne since April, 1898, and enjoys the advantage of having been trained at the Seamen's Hospital, Greenwich, and, for four years, at the Royal Free Hospital. She has had considerable experience as a district nurse, and holds the certificate of the City of London Lying-in Hospital.

### MOURNING FOR NURSES: SUGGESTION BY THE LORD CHAMBERLAIN.

Several of our readers having asked us whether any kind of mourning should be adopted by nurses who wear coloured uniforms, we communicated with the Lord Chamberlain, and have received the following reply from Sir S. Ponsonby Fane :—"The Lord Chamberlain is not authorised to give directions as to the amount of mourning to be worn by nurses. He would suggest, however, that those who wear coloured uniforms might adopt the example of the army and wear a black band on the left arm." The suggestion of the Lord Chamberlain—which coincides with the views of the Council of the Royal British Nurses' Association—admirably meets the case as far as it goes, and we are sure that it will be gladly and generally adopted by nurses indoors who desire to pay a mark of respect to the memory of Queen Victoria. But Lord Clarendon obviously loses sight of the fact that the outdoor costume of most nurses consists of a long cloak which entirely hides the arms. In these circumstances the nearest approach to the Army regulation would be to tack the band, which would otherwise be worn round the arm, straight along that part of the cape which comes just below the shoulder. An alternative, which may be preferred by some, is a rosette of crape pinned on the left of the cloak in front.

### THE TRIBUTE OF THE NETLEY NURSES.

Among the wreaths received at Osborne was one from the Royal Victoria Hospital at Netley, which bore the inscription, "A small token of loyalty and deep regret from Miss Norman and the nursing sisters." It is stated that this beautiful tribute gave special pleasure to the members of the Royal family who recall the deep interest which Queen Victoria took in the work of the Hospital, and the many visits she made to the institution during the progress of the war.

### OVERWORKED ARMY SISTERS IN SOUTH AFRICA.

A few weeks ago we mentioned facts which suggest that the arrangements in regard to the distribution of the nursing staff available in South Africa are still extremely defective. In particular, we drew attention to the fact that five out of six of the Princess of Wales's Nurses were incapacitated in less than twelve months owing to pursuance in the mistaken policy of keeping the sisters continuously at work. An object lesson is supplied in our columns to-day by a nurse on the spot, who, though she does not make any complaint of being overworked in the remarks which follow her bright account of Christmas festivities in hospital, forcibly shows that our insistence upon the urgent need for a better control of the Army Nursing Service at the centre of operations in South Africa is justified by the present situation. Our correspondent is evidently afraid that another typhoid season will tax the physical resources of the nurses who went through the last epidemic, some not unscathed, to the utmost; and she pathetically confesses that after nine to twelve months' work she does not herself feel so fit to enter upon the task as when she started at the outset. Of course she does not, and we trust that the War Office have already given their attention to the question of replacing the sisters who have been through the experience of our correspondent by recruits in vigorous health from home.

### THE SOUTH AFRICAN HOSPITALS COMMISSION AND FEMALE NURSES.

The essence of the report of the South African Hospitals' Commission on the question of female nurses is contained in the following passages :—

> In this war, at any rate at its commencement, some members of the Royal Army Medical Corps appear to have had a difficulty in divesting themselves of the old traditions of the Service, which are undoubtedly antagonistic to the employment of nurses in military hospitals. For example, we find that when the Langman private hospital arrived in South Africa, its principal medical officer applied to the authorities for the assistance of nurses, but his application was refused, though ultimately nurses were supplied to it when it arrived at Bloemfontein. This prejudice, however, has long since ceased to operate in the care of the sick and wounded in South Africa. The nurses employed in this war have shown great devotion, and many have lost their lives in the discharge of their duties. Scarcely any complaints have been made during this campaign with regard to the nurses. The soldiers have much appreciated their services, and seem to prefer to be attended by them to being attended to by orderlies. But, bearing in mind the ordinary conditions of a military hospital, we think that it will always be necessary, even in fixed hospitals and in suitable wards, that the employment of nurses should be supplemented by that of properly trained orderlies.

This is satisfactory so far as the views of the Commissioners on female nurses are concerned. But it confirms the statements that at the beginning of the war nurses were used practically only for the purpose of superintending orderlies, the allowance of eight nursing sisters to a general hospital not permitting of their actually attending the patients. Though this defect was remedied later, and after the middle of January, 1900, all general hospitals left England with twenty nurses, we greatly regret that the existence of a senseless prejudice should have prevailed to such an extent that for the first period of the war—some three or four months—our sick and wounded soldiers were practically nursed by orderlies. The persons responsible for this state of things should be severely called over the coals. With reference to the objection urged against the employment in the war of nurses who were locally chosen, on the ground that they could not be and were not properly qualified or trained, the Commissioners find that the local nurses were carefully selected, "and that they did their duty well." No complaint," they add, "was made against any of them except in three cases. Of these, one was quite trivial; and in the other two the nurses were trained, one in England and the other in Ireland."

### A BELGIAN NURSE IN BRITISH SERVICE.

It will be recollected that Mme. Alice Bron, a Belgian nurse who went to the Transvaal to nurse the Boers, referred in very unfavourable terms to her patients. She has now, according to the daily press, gone back to South Africa to nurse British soldiers, "an exception having been made in her favour to the rule which excludes foreigners from the British hospital service." We assume, this being the case, that her training is satisfactory, and that the reason the rule has been set aside is that Mme. Bron has a special knowledge of South Africa, both as regards the climate and the particular difficulties she will have to encounter, as well as experience in the nursing of typhoid.

### THE RAILWAY COMPANY AND THE ARMY NURSE.

For an offence which the magistrate at Southwark Police Court accurately described "a scandalous and cruel theft," Walter Morgan, a reservist from the King's Royal Rifles, has been justly sentenced to six months' hard labour. The prisoner asked for mercy on the ground of his good character "and military services." But a very dangerous precedent would have been set if Mr. Slade had allowed the consideration of Morgan's military services to weigh with him. A soldier who can rob a nurse is not fit to wear the King's uniform. We are glad to observe that the South-Western Railway Company, though not of course responsible for the loss sustained by Miss Rogers, have decided to compensate her. When her property was recovered by the police it was found that the clothing had been in use, and was in a bad condition. Therefore, but for the generosity of the railway company Miss Rogers would have suffered severely in pocket at a time when she could have least afforded it.

### THE YORK HOME AND THE ALEXANDRA NURSES.

A simple little statement about the Alexandra Nurses in our issue of January 19th has provoked a very indignant letter from Dr. Ramsay, of York. Alluding to the nursing at the stations, Colonel Gildea informed our Commissioner that at York the Soldiers and Sailors' Families Association "work in connection with the York Home for Nurses." As showing that, as we stated, the Soldiers and Sailors' Families Association do work in connection with the York Home for Nurses, we may add that, by an arrangement with the York Home for Nurses, the families connected with the York garrison are attended by nurses belonging to the Home on condition of a quarterly payment of £8 15s. guaranteed by the Council of the Soldiers and Sailors' Families Association. Surely, this is accurately described as working in connection with the York Home. We need scarcely add that there was not the remotest idea of doing any injustice to the excellent organisation whose interests are so successfully, if excitedly, defended by the honorary medical officer.

### ROYAL NURSES' HOME AT SALFORD.

On Saturday Lady Mottram formally opened the Royal Nurses' Home at Salford. The home has been in occupation for about twelve months, but the opening ceremony was unavoidably postponed. The late Sovereign, in response to the application of Sir Richard Mottram, was graciously pleased to authorise the use of the word "Royal" in connection with the title of the institution, which has been erected at a cost of £10,000 to commemorate the Diamond Jubilee of Queen Victoria. The design of the building is substantial and pleasing, and harmonises well with the Royal Technical Institute, on the opposite side of the Crescent.

### A HABIT TO AVOID.

["The nurse arrived, and almost the first thing she said to the patient was, 'Where am I to have my meals?'" Vide B.P.'s letter, Nursing Mirror, January 26th, 1901, pp. 229-30.]

There are certain nurses who apparently think
There is naught in the world like victuals and drink,
For "Victuals and Drink" is the first thing they cry,
And of Victuals and Drink they will probably die.

*A Sufferer.*

We have received the above lines from a correspondent. When nurses show themselves very particular about their personal comforts, or stickle too energetically for the full recognition of their position, it may be well to recollect that the public do not always regard the matter in the same light.

### RULES IN PRIVATE NURSING.

The interesting correspondence which has been going on in our columns on the subject of Rules in Private Nursing may fitly be brought to a close with a word of comment. It is suggested by "Fair Play," who writes on the subject to-day. The main issue, as she implies, really is whether any nurse should be required to sleep in any patient's room. If a "convalescent," male or female, is ill enough to need medicine several times in the night, and other attentions, it is clear that a nurse ought to be on duty, and she should certainly not be the same nurse who is with the patient during the day. It is a wholesome rule of the Nurses' Co-operation that, whenever possible, a nurse should get at least eight consecutive hours out of the sick-room. Conformity to this rule would, generally speaking, prevent the question of sleeping in the room with a convalescent male patient from being raised.

### NO EXPERIENCE IN NURSING.

The latest illustration of the wrong-headedness of Boards of Guardians comes from Ruthin. In spite of the opposition of the medical officer of the Union, and the assurance of the clerk that the Local Government Board would never confirm the appointment of an unqualified person, the Ruthin Board of Guardians have decided, by a majority of 10 to 8, to appoint a nurse who admittedly has had no experience in nursing. The excuse of the majority is that only one application was received in reply to an advertisement. But as the testimonials of the solitary applicant, who is a person of some audacity, showed her inexperience in nursing, it was obviously the duty of the Ruthin Guardians to ignore her application and advertise again. They will have to repeat the advertisement in the end, and it is difficult to understand why they should have deliberately taken up a position which must expose them to ridicule and rebuff.

### BRISTOL NURSES' INSTITUTION.

The annual meeting of the Bristol Nurses' Institution was held last week. Dr. Shingleton Smith, who presided, moved the adoption of the report, and said it was highly desirable that judicious criticism should be made by those employing nurses when there was any need of such criticism.

The reason for this was that medica men heard complaints sometimes of such and such nurses being a trouble He was not speaking of any nurses connected with that institution. There were many people who were nurses and not nurses, who put on a cap and gown, but were not to be compared with those sent out from that institution. They brought discredit upon institutions like that one, and when people had anything to say about nurses they should say so. Those who could state things pleasant should state them, but, on the other hand, if they could state unpleasant things they should state them.

This is a very sensible way of looking at things. The mistake is for either managers of Nursing Institutions, or nurses, to welcome all the compliments they can obtain, and resent any criticisms which are not flattering. The Bristol Institution itself, which had 59 surgical cases last year in the home with six available beds, whose nurses attended 521 cases, and whose managers made both ends meet without attempting to get a profit out of the nursing, is in a happy position.

### AS IT SHOULD BE.

There is no class of the community amongst whom Nursing Associations are more needed than the mining population in the north of England. There are already a few in the pit districts of the counties of Durham and Northumberland. A nursing association for the district of Ashington in Northumberland has just commenced work. The pitmen at the Woodhorn and Lenton collieries and the Ashington mechanics and enginemen have decided to support amongst themselves a fund by contributions of 2d. per man and 1d. per boy each quarter. This will produce about £75 per annum. Promises of support have been received from the lord of the manor, the Duke of Portland, V.C., the Duke of Northumberland, the Ashington Colliery Company, and the local gentry. A commodious building is being secured, and Nurse Malcolm commences duties forthwith.

### THE NEW NURSING HOME AT DUBLIN.

The Royal City of Dublin Nursing Home, whose foundation stone was laid on April 9th, 1900, by Princess Christian, is making excellent progress. When it is completed it will accommodate thirty nurses, each having a separate bedroom. On the ground floor are the hall, the offices, a sitting-room and bedroom for the matron, a dining-room, and a large recreation room for the nurses. The lavatories and baths are in a detached wing, the staircase is fireproof, and the entire building, which is of brick, faced with Ruabon brick and buff terracotta, will be lighted by the electric light.

### NURSES AS ADVERTISING MEDIUMS.

The following advertisement appears in the *Daily Telegraph* of Saturday, January 26th:—

> LADY DEMONSTRATORS.—WANTED bonâ-fide nurses, to wear their own regulation costumes preferred, to introduce advertised article to customers in chemists' shops. Previous similar experience essential.—Write, stating age, wages, references, &c.

Of course no *bonâ-fide* nurses will apply, and the persons who desire to convert nurses into advertising mediums will only get what they deserve if they are deceived by young women pretending to be what they are not.

### COMMISSON ON NURSING FEES.

An important issue was tried at Bloomsbury County Court on Tuesday before Judge Bacon. The Male and Female Nursing Corporation sued Mr. Stickler, a trained male nurse, for the sum of £8 8s. under the following circumstances. Defendant was engaged by the manager of the Corporation as one of its nurses in May, 1898, and agreed to pay the plaintiffs three shillings on each guinea he received from cases which he obtained through their instrumentality as long as the cases lasted. In June, 1899, he was sent to attend a case at Westminster, in which he is still engaged, and paid the commission due on his fees of £2 2s. a week until May, 1900, since which time he had declined to pay. The agreement was subject to a month's notice, and in April last the nurse gave the Corporation notice, and had his testimonials returned to him. Judge Bacon gave judgment for the plaintiffs, and, addressing the defendant, said: "You cannot get out of your liability by giving notice, and you had better not forget that your liability will go on as long as you attend the case." The judgment is clearly in accordance with common sense as well as with law, and the moral for nurses of both sexes is that they cannot be too careful in considering agreements to pay commission on fees before they sign them.

### THE NEW MATRON OF GUY'S.

Miss Sarah A. Swift has been appointed matron of Guy's Hospital. Miss Swift, who since January, 1893, has been lady superintendent of Guy's Hospital Trained Nurses' Institution, was trained at the Royal Infirmary, Dundee. Subsequently she was acting matron at the Home for Incurables and District Nurses, Dundee; ward superintendent at the City Infirmary, Liverpool; night superintendent at the London Fever Hospital; and sister at the Seamen's Hospital, Constantinople. In December, 1890, she joined the staff at Guy's, and was assistant matron from December, 1891, to January, 1893.

### SHORT ITEMS.

The usual winter weekly lectures to nurses at the City Orthopædic Hospital will commence on Friday afternoon at 4.30, and be continued every Friday until the end of March. The lecturers are Mr. Noble Smith, Mr. Chisholm Williams, Mr. John Poland, and Mr. Jackson Clarke. The lectures are free to all nurses.—Nursing Sister Babb, who has been seriously ill at Wynberg, is reported to be out of danger and progressing favourably.—A written examination was held on the 22nd, at West Ham Hospital, Stratford, E., for twelve probationers on a course of the house surgeon's lectures. The three honorary visiting surgeons awarded the first prize to Nurse Medland, and the second to Nurse Loweth.—The life-size statue of Her Majesty Queen Victoria in the vestibule of St. Thomas's Hospital is draped in regal purple cloth, and a wreath of laurel, arum lilies, and lilies of the valley, tied with broad violet ribbon with a black border, hangs on the front of the figure.—The manager of the Scientific Press will be pleased to supply any of our readers with copies of the beautiful portraits of the Queen Mother and Queen Empress which appeared as supplements to The Hospital at Christmas, 1899. The price is 2d. each.—The nurses' entertainment at the London Temperance Hospital, which in ordinary circumstances would have been held last week, has been postponed in consequence of the death of the Queen.—Nurse Greenhow of the Bristol Royal Infirmary has passed the L.O.S. examination.

### Lectures to Nurses on Medicine and Medical Nursing.

By NORMAN DALTON, M.D. London, F.R.C.P., Physician to King's College Hospital.

(*Continued from page* 208.)

### III. FEVER, OR THE PYREXIAL STATE—THE TEMPERATURE—RIGORS.

THE diseased state which is called fever, or pyrexia, is made up of the following collection of symptoms:—First, the temperature of the body is higher than normal. Strictly speaking, any temperature above 98°·4 constitutes fever, but it must be remembered that some people have naturally a higher or a lower temperature than the average, so that variations between 98° and 99° are not, as a rule, important. Then the pulse is quicker than normal, the normal being about 70°, and the breathing is rather more frequent than it should be. At the same time the patient has a dry, furred tongue, becomes thirsty, loses his appetite, and is constipated. The urine is scanty, dark in colour, and very acid, *i.e.*, it turns the blue litmus paper a more vivid red than normal urine does. The skin is hot and dry, and the patient very often suffers from headache and pains in the limbs.

The above symptoms indicate that "fever" is present without showing what disease is causing the fever. It is not necessary that all of them should be present; for as a matter of fact it is only the thermometer which can with certainty tell us that the patient has fever by registering a temperature higher than 98°·4. Still it is useful to know the other symptoms of fever and to remember that before the thermometer came into use doctors had to recognise the presence of fever by noting the quickness of the pulse, the heat of the skin, and the condition of the urine. Each of these symptoms when considered alone is unreliable, but all taken together constitute very sound evidence of fever. In the South African war the breakage of thermometers was so great that at one time there were hardly any at the front, and the doctors had to fall back on the old methods of observation. The individual symptoms of fever require further consideration. Taking the "temperature" first, the actual height to which it rises is, of course, the only safe indication of the amount of fever present. If the temperature reaches 105° or higher the condition is called "hyperpyrexia," and this is always serious. Hyperpyrexia is most often met with in rheumatic fever. In this disease the temperature may quite unexpectedly run up to 107° or higher, and at the same time the patient may become incoherent, or delirious, or profoundly unconscious. Consequently, if in a case of rheumatic fever the patient shows any confusion in his ideas, the temperature should be taken at once, and if it be 104° the doctor should be immediately informed; for the rise from 104° to 107° may be very rapid, and unless the treatment in such cases is most prompt death almost invariably occurs. Hyperpyrexia may occur in other fevers, such as typhoid, typhus, malaria, &c., and even in fevers like measles, which are usually not serious. But in all cases a temperature of 105° should be immediately reported.

In children the temperature may rise suddenly to 103° or 104° without any cause being found. If there be no real cause for it, it subsides as quickly as it rose.

One method of classifying "fevers' is based on the range of temperature during the 24 hours. If during the fever the temperature falls one or two degrees (say from 103° to 101°·8) every morning and rises again one or two degrees in the evening, the fever is called a "remittent" fever. The difference between the highest and the lowest temperature is called the "remission." Typhoid fever when at its height, the fever of septicæmia and of tuberculosis, and one variety of malarial fever are remittent fevers. On the other hand if the temperature comes down to the normal (say from 102° to 98°) once every day, the fever is called an "intermittent" fever. One form of malarial fever is distinctly intermittent; in some cases of septicæmia and tuberculosis the fever is intermittent rather than remittent; and at the end of a case of typhoid the temperature begins to intermit before it becomes normal. If the temperature remains high for several days, then becomes normal for several days, then goes up again and remains high for several days, then comes down again, and so on, the fever is called a "relapsing" fever. There is a special fever, which most often occurs during famines, which is called relapsing fever because its temperature chart presents the above peculiarity; and in some cases of abscess of the brain and abscess of the liver the temperature may be of the relapsing type. In typhoid fever, in Malta fever, and some others a relapse may take place, that is, the patient, after having been through the disease, may in a week or two be taken with it again and go through the whole course of it a second time.

In prolonged fevers such as typhoid nurses are often requested to keep two temperature charts. On one the temperature at two periods of the day (say at 8 a.m. and at 8 p.m.) is regularly put down. This chart is to show the physician the general course of the disease. On the other, the temperature at every sixth or every fourth hour is put down. The object of this chart is to inform the physician of the duration of the higher temperatures during the day, whether any remissions have occurred, &c., and it is used by him as a guide to the treatment of the temperature by cold bathing, &c.

Some febrile diseases come to an end suddenly, the temperature falling in the course of a few hours from 103° or more to normal, while the other symptoms, particularly the pulse, improve. This method of termination is called termination by "crisis." In some cases profuse sweating, or a copious discharge of urine, or some diarrhœa take place while the temperature is falling. Typhus fever and acute pneumonia usually terminate by crisis. If, in any prolonged fever, the temperature falls several degrees rapidly, while the pulse, instead of becoming slower and stronger, becomes weaker and quicker, it is a bad sign; so that the state of the pulse is a good guide as to whether the fall of temperature is beneficial or not. If the temperature fall gradually, so that it takes several days to reach the normal, the disease is said to terminate by "lysis." Typhoid fever, small pox, and many other fevers terminate in this way.

In prolonged febrile diseases it is usual for the temperature to be subnormal for some days after the disease is over. Subnormal temperatures at such a time are therefore of no consequence; but if they occur in the middle of the disease they usually indicate some serious complications. For instance in typhoid fever a sudden fall of the temperature usually indicates that intestinal hæmorrhage has occurred. In diseases in which there is no fever, but in which there is great loss of strength, the temperature may be habitually subnormal.

It sometimes happens that the patient may feel cold and that his skin may be cold, while the temperature inside his body may be raised. This is most often found in the cold stage of malarial intermittent fever (ague) and in the second stage of cholera, which is called the stage of collapse. In these diseases the temperature should always be taken in the rectum, unless the skin be hot. At the beginning of many fevers, also, the patient feels cold while his internal temperature is rapidly rising above the normal.

(*To be continued.*)

### "Visitors for the Unvisited."

By Our Own Correspondent.

On Tuesday afternoon, at 68 Eaton Place, Lady Stapleton introduced the scheme proposed by Miss Annesly Kenealy for visiting the sick in the London hospitals. The majority of speakers were of opinion that workhouse infirmaries were even more in need of visiting than the hospitals, and that there was also an opening in convalescent homes. The scheme, it appeared, would cover visits to the sick in their own homes and co-operation with the Invalid Children's Aid Association. Very little was said about the subject from the nurse's point of view; and there was a good deal about the need of cheering the "misery and loneliness" of the sick in the "dreary wards." The Chaplain of St. George's Hospital said that the wards had been visited by ladies during the past forty years; there was a full list of visitors, and others were waiting for vacancies.

Mr. Adrian Hope said he thought people did not realise what the visits of young ladies meant to the children: the sisters and nurses had no time to play with them, and they looked forward to the visits of the "lidy" with keen delight. Her clothes were their topic of conversation; they discussed her bracelets; ladies should always go to the hospitals in their best clothes. A hospital was very like a convent: it gave great pleasure to sisters and nurses to see the latest thing in hats. The visitor thus supplied an enormous fund of amusement to her fellow-women. He himself felt cheered by it. "The irregular visitor," concluded Mr. Hope, "is better than none at all. From two to four every day the wards are open. Sisters and nurses and children wait, and the visitors *don't* come." In three months, when there were 200 children in the hospital, he had found only six names in the Visitors' Book at Great Ormond Street.

Miss Kenealy stated that she was unable to point to any one great work started by women during the last century. she thought it was time women began. Hospitals, even those for children and maternity cases, were started and maintained by men. She urged the claims of the proposed association, one point of which would be that clergymen and ladies in the country should be able to write to a central body in London when sending patients to the London hospitals asking for a visitor to be sent.

Mrs. Alfred Spender said the idea that one hour a week would suffice was erroneous; every patient needed a quarter of an hour's visit. A sort of stigma attached to those who had no friends to see them on visiting days, and to such the visitor would find it desirable to go again on the days when the patients' friends were admitted. It was not those from a distance, but the very, very poor who were in this case. They might be ill, and even die, without the knowledge of anyone but doctors and nurses. Any lady who visited properly would find out which of the patients were from the country. It might be a good thing if one lady would be responsible for the visitors, not in the sense of control, but of introduction to the sister of the ward where they were to visit; otherwise it was one of the most alarming things to go into a ward. Infirmary visiting, Mrs. Spender urged, was quite different from hospital visiting: the patients were made up of two classes—either chronic patients, who would recover in any case, and those rejected by the hospitals. Infirmaries were nothing like so cheerful as hospitals: "the nurses felt that the patients would either die or get better, whatever they did." No young girls should act as visitors: they would be so easily imposed upon. The committee should not make the best of things, but tell the visitors plainly what the difficulties were.

Lady Craufurd proposed the following resolution (it was seconded by Lady Stapleton and carried unanimously):—"That a society of visitors for the sick be formed to cooperate with the authorities of hospitals and workhouse infirmaries, or any associations for invalids, in visiting and acting in any useful manner in the interests of sick persons."

A Provisional Committee was formed of ladies approved by the meeting. Other speakers were Mr. Ward Cook, Miss Jervis, and Dr. Schofield, who drew attention to the incidental remark of a previous speaker, who spoke of some infirmary nurses as belonging to the "commonest description of pauper." Dr. Schofield said he was in hopes that the training of infirmary nurses had improved.

### Sick Soldiers in South Africa.

### CHRISTMAS IN A HOSPITAL.

"A Happy Christmas, Sister!" What a curious sound to English ears on a glorious summer day, very hot even at 7 a.m. It was hard to realise that it was Christmas in South Africa to those of us who are accustomed to the cold of an English December, but the old familiar greeting was heard on all sides, as we went on duty on Christmas morning, with an accompanying growl as to when would this war be over, &c. Here in one large general hospital of over 800 beds, Tommy had shown great ingenuity and considerable taste in decorating the marquees, and as all the available material was coloured paper, really the results were wonderfully good. One marquee was most tasteful and delicate with festoons of pale green and pink, interspersed with mottoes. These, as in all the other tents, were mostly in reference to Christmas and the Queen. In another a kangaroo and emu had been cut out and placed on the ward table out of compliment to an Australian sister who was in charge of that particular marquee. Tommy's resourcefulness showed itself too in one tent where coloured paper was running out by the utilisation of three young trees of blue gum, which were tied to the tent poles, and were most effective; I never quite realised, I think, what artistic tastes soldiers had before. I gave a coloured calico print of Lord Roberts to one ward, with which they were delighted, but would not rest till a picture of General Buller was pinned underneath; he is just adored by the men. Plum puddings were the great excitement on Christmas Day, and many were the groans amongst my patients—who were mostly convalescent enterics waiting for a convoy to Durban—that they were not allowed any. However, I made up as far as I could for their disappointment by giving them an extra tin of butter for each ward, and plain cake for tea; the latter caused great satisfaction.

### A Tea for the Orderlies.

On the Friday following Christmas Day a big tea was given to the orderlies (by subscription) as was also one for the convalescent patients on New Year's Eve. Both gave much pleasure, and were well worth the endless bread and butter and cake cutting up which we sisters had previously to get through. One orderly had twenty cups of tea, and survived to tell the tale, for he recounted it to me next day with many expressions of appreciation of the tea generally. Tobacco to the orderlies and cigarettes to the patients were included in the ménu, and I think nearly every patient or orderly had some little gift from the sisters of the wards.

### The Constant Work Beginning to Tell.

Our work is now getting almost entirely medical; the enteric season, of course, has begun, and even without the terrible conditions of last year, such as long sieges, &c., I think our hands will be full. The heat is very trying, and most of us who have been out since the beginning of the war are feeling we have not got the work in us that we had nine or twelve months ago. The rain and thunderstorms are tremendous, and make "gum" boots and a macintosh or thoroughly waterproof coat necessities. The difficulty of trying to get into tents at night when it is pelting with rain ought to be tried to be understood. Luckily for us the weather was fine at Christmas, however, the rain has since made up for lost time.

### To Nurses.

**We invite contributions from any of our readers, and shall be glad to pay for "Notes on News from the Nursing World," or for articles describing nursing experiences, or dealing with any nursing question from an original point of view. The minimum payment for contributions is 5s., but we welcome interesting contributions of a column, or a page, in length. It may be added that notices of entertainments, presentations, and deaths are not paid for, but, of course, we are always glad to receive them. All rejected manuscripts are returned in due course, and all payments for manuscripts used are made as early as possible at the beginning of each quarter.**

### The Royal British Nurses' Association.

QUEEN VICTORIA AND THE NURSING VOCATION.

THE Council of the Royal British Nurses' Association met on Friday, at 10 Orchard Street. There was a good attendance of members.

The Chairman, Sir James Crichton-Browne, said that many touching letters had been received by the Secretary, from nurses in different parts of the country, expressing their feelings of sorrow and sympathy with the President, H.R.H. Princess Christian, in her great loss. Never before had so many human heads been bowed in unanimity of sorrow; never had so many human hearts been touched simultaneously by a sense of personal bereavement. The Queen, unlike any Queen who had gone before her, had no enemies, for even those who had fought against us had paid homage to her sweet and noble personality; she would remain henceforth for ever enshrined in our memories, the ideal monarch of our race, a bright ensample of royal dignity, of womanly tenderness, of simple piety and noble serenity of soul. Amidst the general prevailing mourning, it was fitting that the Royal British Nurses' Association should record its special grief, since it had enjoyed the favour of the late Queen, and had been presided over from the first by one of her daughters, who inherited many of her gracious qualities.

NURSING A CREATION OF THE VICTORIAN ERA.

"Nursing," the Chairman went on, "is a creation of the Victorian Era. From coarse and ignorant empiricism it has grown into a science and an art; during the last fifty years, and at every stage of its growth, it has been fostered by Royal favour. It was because the Queen recognised the good work which this association is capable—if wisely guided—of accomplishing in still further improving the science and art of nursing, and so in alleviating suffering, that she permitted it to assume the prefix "Royal." At every opportunity she had shown her interest in the nurses' mission, and almost the last public duties she performed were sick-bed ministrations, visits to wounded soldiers home from the war, who felt almost compensated for their pains by her kind looks and words.

THE RESOLUTION OF SYMPATHY.

"Nurses, in spite of their calm exterior," continues Sir James, "have sensitive and sympathetic natures—they could not be good nurses if they had not—and I am sure that every member of this Association is in genuine sympathy with our Royal President in her poignant sorrow at this moment. We know with what unwearying filial devotion she has waited on the Queen in recent years, even when anguished by the calamitous death of her brave son; and having ourselves felt the brightness of her presence, we can understand how she has cheered and illumined her mother's declining days. She has the consolation of many radiant memories. Unless I am much mistaken she will find further consolation in her renewed participation in the benevolent works with which her name is associated. Perhaps she may find some small consolation in the humble but heartfelt message of condolence which we propose to send to her."

The Chairman then submitted a resolution of sympathy with Princess Christian in her irreparable bereavement, which was seconded by Mrs. Coster, and supported by Mr. Langton and Mr. Fardon, who said: "We know that our President would not like to feel that the work had been hindered in any way by the decision to adjourn the business of this meeting; but we are not in any way hindering the affairs of the Association, because there was not any business which needed to be done at once." The Council rose, and remained standing while the resolution was carried.

Several nurses had written with the desire to know whether they should wear any sign of mourning on their uniform, and after some discussion, it was agreed that members of the Association should be recommended to wear a band of crape on the left arm during the time the public mourning continued. The meeting then adjourned.

### A Bit of Black Ribbon.

IT'S only a bit of black ribbon,
For mother's poor, you see;
But she's bought a new black hat for herself,
And a bit of ribbon for me.

I'm only a little girlie,
Yet I've seen our dear Queen twice,
And oh! she had a sweet, kind face,
And looked so good and nice.

My daddy went away to the war,
Right across the sea,
And the only thing he was sorry for
Was leaving mother and me.

And mother dreaded Christmas,
She said she hated the day:
It made her feel so miserable
With daddy far away.

But the good Queen thought of the children,
And gave us a lovely treat—
A beautiful Christmas tree we had,
And such nice things to eat.

And the Good Queen came and spoke to me,
And I wasn't frightened at all,
For she was as kind as kind could be,
And gave me a lovely ball.

Next time I saw our Gracious Queen
'Twas at the hospital gate,
Where we'd gone to see Daddy, come home so ill,
But they said we had to wait.

For our Gracious Queen was talking to him;
And while we waited outside,
She came right out, and passed us close,
And when mother saw her she cried.

For she said that there never had been such a Queen,
And that nobody ever could tell
How much she had done for the poor sick men
To help to make them well.

'Tis only a bit of black ribbon,
But I wear it just to show
That I am so sorry our dear Queen's dead,
Because I loved her so.

I. GRIFFITHS.

### Appointments.

INFECTIOUS HOSPITAL, RUSH GREEN, DAGENHAM.—Miss Mary A. Baynes has been appointed matron. She was trained at Cheltenham General Hospital, and St. John's Home, Norfolk Street, Strand. Subsequently she was matron at Sandhurst Hospital, Waltham Abbey.

ABINGDON COTTAGE HOSPITAL.—Miss Mary L. Pollett has been appointed matron. She was trained at the London Hospital, and has since been ward sister at Radcliffe Infirmary, Oxford, and assistant matron at Nottingham General Hospital.

DOVER HOSPITAL.—Miss Florence Brough Law has been appointed matron. She was trained at the Royal Infirmary, Glasgow, and has since held the appointments of lady superintendent of nurses at the Glasgow Royal Infirmary, lady superintendent of the Kent Nursing Institution, Bromley, and lady superintendent of Bromley Nursing Private Home.

### Ophthalmic Nursing.

By a Sister.

### II.—APPLICATION OF REMEDIES.

It will naturally be understood by any trained nurse that the strictest antiseptic precautions must be observed in the application of all dressings to the eye. Therefore, it is unnecessary to dwell largely upon that subject, but on the actual administration of remedies, such as lotions, drops, or ointments, &c., a few hints to those nurses who have not had much experience in ophthalmic work may be acceptable. The golden rule with these remedies is to remember that they must go into or on the eye, not on the edges or outside of the lids. The application has not, as the uninitiated imagine, been suitably made by the patient leaning over a basin of lotion and freely bathing the closed eyelids.

### Lotions.

If the eye has to be washed out with lotion, direct the patient to throw his head well back. With the finger and thumb of the left hand open the eyelids, taking particular care not to press on the eye with the finger when lifting the upper lid; with the free hand dip a swab of absorbent cotton-wool into the lotion and allow it to stream into the eye, seeing that the force of the flow is broken by directing it through the upper eyelashes. The lotion can be received by a large piece of wool or a kidney-shaped receiver held closely to the patient's cheek. This is a most effectual way of thoroughly cleansing the eye; but some oculists prefer having a small glass irrigator used.

### Drops.

Drops are most frequently used to cause dilatation or contraction of the pupil, so it is an easy matter for the nurse to learn the effect of the different drugs by watching the changes in the eye after the application. They are also used for numerous other purposes. The nurse must draw the lower lid down, the patient at the same moment looking up; one drop of the fluid is allowed to fall into the eye by means of a drop-bottle specially designed for the purpose. Care must be taken not to allow the nozzle of the bottle to touch the eye. Should this accidentally occur, the nurse must disinfect the bottle, preferably by sterilisation.

The putting in of drops is generally a simple business with adults; but with children it is very often impossible to manage without the aid of another person to hold the child in a horizontal position, while the nurse, seated opposite, holds the child's head between her knees. This leaves both hands free; so, gently opening the lids, she can administer the remedies. Whilst on the subject of the management of children I might caution the nurse in all cases where there is a large quantity of discharge present, but more especially in cases of ophthalmia neonatorum, to keep her own face well away from the affected eyes, as sometimes when the lids are first opened the discharge is apt to spout upwards and thus affect the eyes of the examiner.

### Painting the Lids.

In these purulent cases and in many others, the surgeon may order an astringent to be painted on the inner surface of the lids. This can only be done by first everting the lid. It will greatly simplify the eversion of the upper lid if the patient can persistently look down. The nurse will then, holding the eyelashes, draw the lid downwards, and dexterously turn it up, using a finger to act as a pivot. This with practice can be done expertly and quite painlessly, but the beginner may find it a help to use a probe to twist the lid round on instead of her finger. A small camel-hair brush should then be dipped in the liquid and drawn over the entire exposed conjunctiva. The eversion of the lower lid can be accomplished by the patient looking up, the nurse simultaneously drawing the lid downwards.

### Ointments.

A small glass rod narrowing at the point is an excellent instrument for inserting ointment with, but in lieu of this an ordinary silver probe can be used. A minute piece of the ointment is taken on the end of the instrument, which is then horizontally inserted between the globe and the lower lid, the patient involuntarily squeezes the lids together, and the probe can be withdrawn at the outer canthus leaving the oinment in the eye. If the cornea is the affected part, a light circular movement of the finger on the closed upper lid will diffuse the ointment over it.

### Fomentations and Cold Pads.

A good method of applying a fomentation is to get a pad of absorbent wool, over this sprinkle some boric acid powder, put it into a towel and wring it out of *boiling* water. Then give the wool a shake to make it light and fluffy, and apply to the eye as hot as the patient can bear, and change when cold. Cold applications are made by dipping a round pad of gamgee tissue lightly into iced lotion. This should be tied on with a thin bandage and changed every twenty minutes.

### Powders

Powders are not used so much in eye dressings as ointments, but when they are ordered the nurse will find a neat way of applying them to the surface of the eye, is by taking a little of the powder on a small camel-hair brush, and having opened the patient's eye, she can hold the brush between the thumb and second finger, while a tap with the fore-finger on the brush will flick the powder where it is required.

In dressing eyes it is better, when it is possible, for the patient to face a window, as this enables the nurse to see if any change has taken place, but she must always be guided in this by the nature of the disease. In some instances, where there is much photophobia present, it is positive cruelty to open the eyes in a strong light. It is always a wise proceeding to modify the light in all cases where intolerance of light is strongly marked. For instance, when the patient is suffering from corneal ulcers he will often bravely try to open his eyes, if he feels assured that the room is "nice and dark," but as long as there is a bright light the eyes will be as tightly closed as oysters.

### Minor Appointments.

King's Norton Union Infirmary.—Miss Nellie Woodcock has been appointed charge nurse. She was trained at the Chorlton Union Infirmary.

Kindray Hospital, Barnsley.—Miss A. Mattinson has been appointed charge nurse. She was trained at the Royal Infirmary, Manchester, and has since been staff nurse in the same institution.

Marlboro' Workhouse Infirmary.—Miss Emma Tull has been appointed assistant nurse. She was trained by the Meath Workhouse Nursing Association, and for the last year has been assistant nurse at the Whitchurch Union, Hants.

St. Saviour's Infirmary, East Dulwich Grove.—Miss Frances Wallen and Miss Janette Hughes have been appointed sisters. Miss Wallen was trained at the Metropolitan Hospital, Kingsland Road, and also holds the L.O.S. Miss Hughes was trained at Guy's Hospital.

Woodford Jubilee Hospital.—Miss Parsons has been appointed staff nurse. She was trained at the General Infirmary, Chester. She has since been nurse at the Norfolk and Norwich Hospital, and has done private nursing as a member of the staff of the Croydon Nurses' Institute.

St. Saviour's Infirmary, East Dulwich Grove.—Miss Margaret Elliott has been appointed night superintendent. She was trained at the Western Infirmary, Glasgow, and has since been sister-in-charge of the Lady Hozier Convalescent Home, Lanark and sister at St. Saviour's Infirmary, East Dulwich Grove.

### Echoes from the Outside World.

### AN OPEN LETTER TO A HOSPITAL NURSE.

THE week which has elapsed since last I wrote to you has been one of the strangest through which I have ever passed. Though one has, of course, often alluded to the transitoriness of human things in the words, " The King is dead, Long live the King," it had never dawned upon most of us that it would fall to our lot to mourn the death of a Queen one day with tolling of bells, and flags half-mast high, and to hear her successor proclaimed the next, with cheers of " God Save the King," whilst the flags, suddenly run up, fluttered in the breeze. But such things needs must be, and though for a few hours on Thursday the nation, as it were, tried to forget its grief in giving a welcome to the son of his mother, it was only a passing gleam of brighter things. On every side the down-drawn blinds of private houses, the black boards or the lowered shutters of the shops, and the forsaken theatres tell the same sad story. Here and there some efforts have been made at mourning decoration, black draperies relieved by violet or white being arranged over some of the larger emporiums, but to my mind anything which savours of sensational display is to be avoided, and all should be as simple as the life and tastes of her whom we mourn.

THE meeting of the Houses of Parliament on Friday forms part of our national history, and as such possesses exceptional interest for us all. The necessity for what some have thought the premature proclamation on Thursday, when the heralds at St. James's Palace, at Temple Bar, and the Royal Exchange declared the erewhile Prince of Wales King Edward VII., was apparent the next day when Edward Rex as monarch sent a message to both Houses, in which he announced the "lamented death of his beloved mother, the late Queen," and asked for their sympathy in the deep sorrow which had befallen His Majesty and the nation. In the House of Lords, Lord Salisbury, with bowed head and grief-stricken face, moved that an address of sympathy should be presented to the King, with respectful congratulations upon his accession to the throne, and an assurance " of our loyal attachment to his person," and "of our earnest conviction that his reign will be distinguished, under the blessing of Providence, by an anxious desire to maintain the laws of the Kingdom and to promote the happiness and liberties of his subjects." Then the Premier went on to say that in making this motion he had to perform the saddest duty that had ever befallen him, and in speaking of Her Majesty's many titles to admiration said "that one which, I think, will attach to her character in history is that, being a Constitutional Queen with restricted powers, she reigned by sheer force of character, by the lovableness of her disposition over the hearts of her subjects, and exercised an influence in moulding their characters and their destinies which she could not have done more had she had the most despotic power in her hands. She had been the greatest instance of government by example and by love." Lord Salisbury went on to eulogise the high qualities displayed by the Queen in affairs of State, and to state how deep was her insight into political matters, and how no minister ever disregarded her advice afterwards without feeling that he had incurred a dangerous responsibility.

IN the other House the Leader of the House of Commons made a speech which will be long remembered and frequently quoted. Mr. Balfour was not so overcome in speaking on the subject of Queen Victoria as Lord Salisbury, probably because his life has not been bound up with her in the same way. In the happiest words, Mr. Balfour touched on the effect which the character of the Sovereign produced upon all who were brought in contact with her. " Her simple dignity, befitting a monarch of these islands, could never fail, because it arose from her inherent sense of the fitness of things. It was no trapping put on for office, and therefore it was that this dignity, this queenly dignity, only served to throw into higher relief and into a brighter light those admirable virtues of the wife, the mother, and the woman with which she was so richly endowed." "Perhaps," he continued, "less known was the life of continuous labour which her position as Queen threw upon her. Short as was the interval between the last trembling signature affixed to a public document and the final rest, it was yet long enough to clog and hamper the wheels of administion, and I remember, when I saw the vast mass of untouched documents which awaited the hand of the sovereign of this country to deal with, it was brought vividly before my mind how admirable was the unostentatious patience with which, for over sixty-three years, through sorrow, through suffering, in moments of weariness, in moments of despondency it may be, she had carried on without intermission her share in the government of this great Empire." I feel glad that Mr. Balfour emphasised this last point. One is too apt to forget that a reigning monarch has much business to do which is neither social nor political, and the reminder may help to make us more considerate in the future towards our new king and his august consort, whom Sir H. Campbell-Bannerman said truly had reigned in the hearts of the British people ever since she set foot upon our soil.

THE question of mourning for Queen Victoria is of far greater moment to women than to men, because a hatband and a black tie are the only additions which the ordinary male requires, in conjunction with his black coat and waistcoat, to show that he has lost someone who is dear to him. The mourning for nurses in uniform is dealt with by the Lord Chamberlain; but as many of you leave your dress as well as your duties behind you when you go for a holiday, the probability of wearing black or modified black for a year necessarily caused some perturbation, more especially to those whose purse is of modest dimensions. The official announcement of the modification of the orders for mourning which the Earl Marshal made on January 28th, that the general public should wear deep mourning till March 6th, and then half mourning till April 17th, has been a great relief to all. The boon has, of course, been principally conceded for the sake of the wholesale drapery trade, some of whom would have been ruined if all their light spring and summer goods had been thrown on their hands—the inevitable result if the order had remained in force till the 24th of January next year. The most striking feature of the mourning is that before any injunction to the public had appeared, perfectly spontaneously and out of real love for her Majesty nearly everyone who could possibly do so had already donned black, or made arrangements for doing so. Even the children of ten or over who are not in black have some distinguishing mark to show that they, too, mourn the Good Queen; and the appearance of the interior of the churches and the solemnity of the services on Sunday last must have impressed even the smallest children in a way they are never likely to forget. Thirty thousand people, it is calculated by Archdeacon Sinclair, were turned away from St. Paul's alone, and every place of worship throughout the Metropolis was crowded.

THE King has commanded that Saturday shall be a day of general mourning. It would have been so to all of us had never a word been said, but the order will prevent any awkwardness in business matters. Thousands will throng the streets in order to pay the last act of homage they can to her whom they held so dear. Many more will line the sides of the Solent and the embankments of the railway from Portsmouth to London, and yet thousands upon thousands will repair quietly to their respective churches and thank God for the past and pray for the future. Those who are desirous of seeing the funeral pageant in London will have to be up betimes, for the route will be kept even more carefully than at the Jubilee of 1897; and when standing-room is exhausted, the avenues of approach will be blocked, and no one will be allowed to pass into the thoroughfare. Owing to the limited amount of shop window accommodation along the line of route, seats have fetched quite extraordinary prices, while the few which remain unsold are likely to be priced still higher; but it is very much to the credit of some of the large shops who hold the Royal Warrant that they have decided to close their shops, and out of respect to their late Sovereign forego the harvest they might have reaped.

### Nursing on the "Aurania."

### A CHAT WITH A SISTER OF THE NEW ZEALAND RESERVE.

By a Correspondent.

The *Aurania*, a magnificent ship of the Cunard Line, brough about 670 invalids, many serious cases, both wounded in battle and enteric, in fact, "all sorts," as the P. M. O. told me, but they had "no deaths on board amongst the troops, whose general health had wonderfully improved on the voyage." Most of the men belonged to the 14th and 18th Hussars and Highland Infantry.

Sister Redstone, who was trained in Victoria, was doing "special" on a patient who had been shot in the head: he had had more than one terrible operation, and, altogether, sixty pieces of bone had been taken from his skull; his wound had produced partial paralysis, which necessitated his receiving extra nursing and care. Some other patients had also at one time shared her nursing, but these were, happily, convalescent.

Just before the ship left South Africa several Naval Reserve men were put on board; one poor fellow in the last stage of phthisis succumbed two days afterwards.

When asked whether she was returning, Sister Redstone answered brightly, "Oh yes, you see I belong to the Government; I quite long to be back, but I am very glad to be here for a short holiday, as I have been ill myself. You see I have been nine months in South Africa."

"Did the announcement about our Queen make any effect on the men?" I inquired.

"Yes, indeed; a gloom was thrown all over the ship—we learnt the news at sea."

"Are the sisters really short of clothing, &c., at the front?"

"No," emphatically was her reply. "They can get anything that they want, and the tales that are carried about by some people are absurd."

Sister Redstone herself was attired in a most useful and becoming costume, the uniform of the N.Z.R.—a khaki cloth dress, with a three-quarter coat to match, red collar and red bow at neck; white apron and regulation collar and cuffs and cap when on duty. I expressed my surprise at seeing no bonnet, but a brown felt hat is worn out of doors. And as Sister Redstone said, "a hat is much more serviceable for the rain and storms of sand, and more suitable for working in tents." Two dresses, she added, are allowed each sister per annum; and khaki linen dresses are worn in surgical wards."

### Age 18—81.

Same figures, yet suspense between
The reign of our belovèd Queen.
Same figures, yet between them lie
The grandest reign in history.
Pattern mother, pattern wife,
A queenly Queen throughout her life;
Sharer of every hope and fear,
Growing much dearer year by year.
Her country's hope, her people's friend,
Aiming with justice love to blend.
Same figures, yet between them trod
A gracious sovereign child of God.
But, like the prophet, she hath flung
Her mantle on our King, her son.

—*B. K. Coles.*

### The Nurses' Bookshelf.

Manuals of Employment for Educated Women. Edited by Christabel Osborn. Vol. I., Secondary Teaching. Vol. II., Elementary Teaching. (London: Walter Scott.) Other volumes in preparation.

Although women are fast waking up to the fact that the two essentials of success in any profession are suitability and adequate training, the unfortunate tendency to drift into an employment without due regard for either still exists. It is with a view to checking this tendency, by giving definite information about various branches of women's work and the necessary qualifications for them, that these useful little books have been prepared: they are handy and concise, and the cost of one shilling each is certainly not excessive. It is perhaps natural that the teaching profession should be considered first; many facts prove that it is largely going into the hands of women, so much so that in America, in certain districts (to quote the introduction by Miss E. P. Hughes), "it has become a serious educational problem whether man teachers will not completely disappear." On the other hand, the tendency towards mixed schools with men teachers, which some educationalists recognise, may possibly restore the balance. Vol. I. deals with "Secondary Teaching," the function of which public opinion in England has at present failed to grasp, and which is, consequently, in so chaotic a condition. Full particulars of how and where to train for posts in girls' schools, colleges, and as private governesses and coaches are given, and the advantages and disadvantages of the life of a teacher are fairly faced. The chief drawback—and one by no means peculiar to this profession—is undoubtedly the uncertainty as to the future: "I often wonder," says one lady quoted, "what I am going to do at fifty, when my services will no longer be required at the high school. Perhaps by that time a universal pension scheme will have come into existence." It is just here that the teacher in the elementary school has the pull over the secondary teacher—her future is assured, though her pension be small. Here, too, is a greater demand, and consequently more choice as to locality, &c., while the secondary teacher must take what she can get. Vol. II. shows that there is a wide field of development for educated women in elementary schools. "Variety of type," says Sir Joshua Fitch in the introduction, "is essential in so comprehensive a national system as our own." It is one of the few professions not overstocked, and its importance as an opening cannot be denied. The present proportion of women to men, as elementary teachers, is as follows:—Women, 80,057; men, 28,978; girl pupil teachers, 26,706; boy pupil teachers, 6,643. This volume gives full particulars of how and where to train for posts in primary schools, including schools for the afflicted, Army schools, industrial and Poor Law schools, evening continuation schools, pupil teacher centres, training colleges, and higher appointments under the Board of Education and School Boards.

### Presentations.

Guy's Hospital.—The sisters, nurses, and others of Guy's Hospital, have presented a handsome silver coffee service to Miss E. H. Young, together with an address, expressing their regret at her resignation of the post of matron, and their appreciation of her work during the time she has been amongst them.

Hitchen Union Infirmary.—Miss Mary Jarvis, on resigning her post of superintendent nurse (which post she has held for the past six years), was presented with a biscuit barrel and timepiece made of walnut with silver mountings and monogram in silver from her fellow officers, and an inkstand to match from the patients, in token of the great esteem in which she was held.

### Everybody's Opinion.

[Correspondence on all subjects is invited, but we cannot in any way be responsible for the opinions expressed by our correspondents. No communication can be entertained if the name and address of the correspondent is not given, as a guarantee of good faith but not necessarily for publication, or unless one side of the paper only is written on.]

### SO-CALLED TRAINED NURSES.

"A Male Nurse and Masseur" writes: In reply to your correspondent "M. M. M." surely his must be an isolated case, as no sane person would employ a man and send him out without having ample evidence of his having had at least two years' nursing experience. I for one protest against such letters being published, as they are apt to damage those who have had the training, and are fully capable of nursing any case (male), either surgical, medical, or mental.

### "A BRISTOL SCANDAL"

The Lady Superintendent of the Bristol District and Private Nurses' Home, 6 Berkeley Square, Clifton, writes: Referring to a paragraph in your last edition headed "A Bristol Scandal," may I say that the untrained nurse now working in the parish of St. John, Bedminster, is not connected with the Bristol District Nurses' Society, and that the instruction given to her by the late nurse was not done with the knowledge and consent of the committee, who are anxious that only nurses with *at least* one year's training in a general hospital should be employed in nursing the sick poor. The committee most reluctantly withdrew the nurse from a district where one is so badly needed, and only decided to do so after every effort had been made to obtain funds to carry on the work.

### HOTEL NURSING.

"L. P." writes: In reply to B. P.'s question "Why will nurses be so foolish?" I answer, like a true Irishwoman, by asking another question, Why will nurses criticise their sisters-in-nursing with patients? B. P. is no doubt right on principle, but one story is good till another is told, and the tone in which a thing is said may alter it very much. It is easy to give advice afterwards, and no doubt the nurse who left the gentleman would write, when calm, advice just as sound as B. P.'s. But to return to my question, I think we ought to stick to each other, and instead of siding with patients when they criticise a nurse, try and let them see that nurses are only human, and have faults and feelings like other people. We are not all gifted with perfect control of temper, but at least there ought to be honour among thieves.

### RULES IN PRIVATE NURSING.

"Nurse Catherine" writes: I have been much interested in the correspondence lately appearing on this subject. As far as my experience goes, when the convalescent stage is reached the doctor's expressed opinion is that the patient should not be aroused in the night from sleep to take medicine. As to the nurse sleeping in a convalescent man's room, where there is only one nurse in attendance she should do so. I have had to do it, and I believe the convalescent stage has its peculiar dangers, particularly the earlier convalescence of typhoid fever. A nurse ought not to feel it an indignity to do so. Of course no nurse would be expected to undress in her male patient's room, and most nurses would prefer to lie on the couch made up for her in her dressing-gown to be on the spot to attend to her patient. She need lay herself open to no charge of immodesty on this account. Of course patients differ; but to speak for myself I have found patients as a rule, and their friends too, more contented to have the nurse close at hand. Knowing that the nurse's rest cannot be unbroken during her attendance on her case, they will as a rule make provision for her comfort during the day that she may have a little sleep.

"Fair Play" writes: Touching "L.'s" letter as to a nurse sleeping in a male patient's room, the question occurs to me, Is it right for a nurse, in duty to herself, to sleep in any patient's room? What other class of worker is there who would be "on duty" day and night? Of course, anyone worthy of the name would not object to extra duty at a crisis, but when convalescent I contend that the nurse should be allowed a reasonable amount of rest to "pick up" for the next case. People often forget that we go from one case to another. I belonged to a provincial home for three years, and was often sent straight from one case to another. The home was very comfortable, but we were seldom in it. If we could rest for a week between cases it would not so much matter if we got no undisturbed rest while at work. I am now on a co-operation whose rule is that whenever possible the nurse is to get at least eight consecutive hours from the sick-room. And quite right, too. The public (that portion which does not know) should be taught that the presence of one nurse in the house does not leave other persons free for twenty-four hours. If two are at work it is a different matter.

### MISS TWINING'S LETTER.

"An Old Hand" writes: I also have read Miss Twining's letter with great interest. The subject in question is one which, for some time, I have wished discussed. I cannot but think that many good and capable women have been lost to the nursing profession simply through lack of proper "handling" in the early days of her probation. What high-spirited young girl can stand being bullied, or what highly-strung, sensitive young woman being harried and worried, by an impatient, inconsiderate teacher? New nurses are often too anxious to do the right thing, and suffer most keenly in the hands of an intolerant upper nurse. Imagine being asked not to "stand snorting there" when you were simply engaged in your duties. A woman cannot have a true love for her work if she has not the patience to teach those under her care. She ought not to forget the fact that at one time she was a "pro" herself, and ranked amongst the ignorant of her profession. As an old hand I speak feelingly, and hope this may meet the eye of those who have the high and noble duty of teaching others that work in which patience, endurance, sympathy, and tolerance should be all-important.

### THE YORK HOME FOR NURSES.

Dr. James Ramsay, honorary medical officer to the York Home for Nurses, writes from 37 Monkgate, York: In an article, by your "Commissioner," entitled "The Alexandra Nurses," at page 211 of your issue of January 19th, it is stated: "At York, where we work in connection with the York Home for Nurses, there were 89 cases and 1,171 visits." As this statement is absolutely without foundation and is calculated to lead to erroneous impressions, I shall feel obliged by your taking the earliest possible opportunity of correcting it. Your Commissioner has evidently been misinformed. "The York Home for Nurses" has been in existence for thirty years, and throughout that time has supplied nurses in all parts of the city and neighbourhood for gratuitous attendance on the sick poor. At present five district nurses in connection with it devote their entire time to this work, and the home is prepared to extend these operations indefinitely in accordance with the means placed at their disposal by the benevolent. I beg to repeat that the "Alexandra" Nurses do not work in connection with our institution. It is true that the "Alexandra" Nurses are, I believe, most efficiently represented by two of their number, who work in three parishes on the outskirts of the city, and are supported by a committee of three parishes. All such useful organisations are dependent on the generosity of the public, and it is not a justifiable proceeding on the part of those who may be interested in forwarding a recently-established work of this kind to represent (or misrepresent) it as being "in connection" with an old-established institution. Surely it savours of "doing evil that good may come"! For the motives underlying such an action are presumably associated with the desire to increase the share of public support for the newer enterprise at the expense of those who have been pioneers in nursing the sick poor, who have borne the "heat and burden of the day," and whose dearest wish it is to be encouraged in every way in their efforts.

### For Reading to the Sick.

### IN THE HOUR OF DEATH.

THERE is no death! What seems so is transition:
This life of mortal breath
Is but a suburb of the life elysian
Whose portal we call Death.—*Longfellow*.

Oh! call it not death—'tis a glorious rest.
"Yea," saith the Spirit, for all such are blest;
"They rest from their labours;" their work is done,
The goal is attained, the weary race run,
The battle is fought, the struggle is o'er,
The crown now replaces the cross they bore;
The pilgrimage path shall no more be trod,
A rest remains to the people of God!—*E. E. H.*

The flowers of spring may wither, the hope of summer fade,
The autumn droop in winter, the birds forsake the shade;
The wind be lull'd, the sun and moon forget their old decree,
But we in nature's latest hour, O Lord! will cling to Thee.

*Bishop Heber.* 1783.

Death is not death if it raises us in a moment from darkness to light, from weakness to strength, from sinfulness to holiness. Death is not death if it brings us nearer to Christ, who is the fount of life. Death is not death if it perfects our faith by sight and lets us behold Him in whom we have believed. Death is not death if it gives us to those whom we have loved and lost, for whom we have lived, for whom we long to live again. . . . Death is not death; for Christ has conquered death for Himself, and for those who trust in Him.

"Blessed are the dead which die in the Lord; for they rest from their labours, and their works do follow them." They rest from their labours. All their struggles, disappointments, failures, backslidings, which made them unhappy here, because they could not perfectly do the will of God, are past and over for ever. But their works follow them. The good which they did on earth—that is not past and over. It cannot die. It lives and grows for ever, following on in their path long after they are dead, and bearing fruit unto everlasting life, not only in them, but in men whom they never saw, and in generations yet unborn.

*Charles Kingsley.*

"I am the Resurrection and the Life; he that believeth in Me, though he were dead, yet shall he live; and whosoever liveth and believeth in Me, shall never die." Wherever faith in Christ is, there is Christ Himself. There our Lord vouchsafes to stand, though unseen—whether over the bed of death or over the grave: whether we ourselves are sinking or those who are dear to us. Blessed be His Name! nothing can rob us of this consolation: we will be as certain, through His grace, that He is standing over us in love, as though we saw Him.—*J. H. Newman.*

The road to death is life, the gate of life is death;
We who wake shall sleep, we shall wax who wane:
Let us not vex our souls for stoppage of a breath,
The fall of a river that turneth not again.

Be the road short and be the gate near,—
Shall a short road tire, and a strait gate appal?
The loves that meet in Paradise shall last out fear,
And Paradise hath room for you and me and all.

—*Christina Rossetti.*

### Notes and Queries.

**The Editor is always willing to answer in this column, without any fee, all reasonable questions, as soon as possible.**
**But the following rules must be carefully observed:—**
**1. Every communication must be accompanied by the name and address of the writer.**
**2. The question must always bear upon nursing, directly or indirectly.**
**If an answer is required by letter a fee of half-a-crown must be enclosed with the note containing the inquiry.**

*A Question of Notice.*

(198) I was engaged by the week as mental nurse to a patient residing in the house of a medical man. Her friends made arrangements for her to be removed to an asylum, and, without my consent, for me to accompany her. I gave a week's notice as I did not wish to do so. Two days from the date of my notice the patient was removed, and my salary was paid up to the date of her removal, not up to the date of the termination of my notice. Can I legally claim the balance?—*Joan P.*

As you gave notice, and as you had the option of accompanying the patient and completing your term of service, it was quite fair to pay you only for the attendance rendered, and not for that you refused to give.

*Dispensing.*

(199) Would you kindly give me the address of the school in London (mentioned some time ago in THE HOSPITAL), where girls are taught dispensing?—*A Constant Reader.*

There are several. You probably refer to the school of the Pharmaceutical Society in Bloomsbury, W.C. Apply to the secretary.

*Epileptic.*

(200) Will you kindly tell me of any home where an elderly lady suffering from epileptic fits could be placed on payment of a small sum quarterly? What is the lowest charge?—*E. A. P.*

The Meath Home of Comfort, Godalming, Surrey. The charges are most moderate. Write to the secretary for terms, which are adapted to different classes of inmates.

*Introductory.*

(201) I am about take up private nursing, and should be glad to know how to introduce myself to new patients and their friends.—*A Junior.*

Have a professional card printed, and call upon the doctors in the neighbourhood to see if they can utilise your services. A brass plate might also assist you.

*Massage.*

(202) Can you recommend me a cheap teacher of massage—one who is thorough, and who would give certificate?—*M. R. D.*

The Secretary, the Society of Trained Masseuses, 12 Buckingham Street Strand, W.C., could assist you to select a well-qualified and inexpensive instructor.

*Shortsighted.*

(203) Is short sight detrimental to one eager to enter the nursing profession?—*Anxious Inquirer.*

Not necessarily so, moreover the defect can be relieved by spectacles.

*Obtaining Patient.*

(204) After seventeen years' nursing I am taking a small house in a healthy country place, where I desire to receive a chronic invalid at very moderate terms. Slight mental case not objected to. Please tell me the best way of obtaining a p tient.—*L. A.*

Your best plan is to advertise. You will frequently see inquiries in this column for suitable cheap homes for elderly delicate folk. Write also to the Secretary, The After-care Association, Church House, Dean's Yard, Westminster, S.W.

*Address.*

(205) Will you kindly tell me the address of the Hollond Nursing Institutes abroad, especially Paris?—*A. H. S.*

The Hollond Institution has been reconstructed as the Nice Nursing Institute, Villa Soleil, Avenue Thiers, Nice. Write to the Secretary for fuller information.

*Surgical Aid Society.*

(206) Mrs. A. would be glad of the address of an institution where surgical appliances, such as wooden legs, can be bought for poor people at reasonable charges.

The Surgical Aid Society, Salisbury Square, Fleet Street, E.C.

*Qualification.*

(207) Would you kindly let me know if a two years' surgical certificate from the Poplar Hospital for accidents is recognised by the Local Government Board?—*E. P.*

It would probably be recognised if supplemented by approved training in general nursing. The best plan is to write direct to the Secretary of the L. G. B. before making final arrangements.

*Uniform.*

(208) Will you tell me what uniform is worn by nurses at the front?—*Terapia.*

The Army Nursing Sisters wear their own uniform: grey circular cloaks and bonnets, grey beige dresses, white caps tied under chin, and white aprons. They also wear little scarlet capes. The Reserve wear navy blue cloaks with red facings, blue bonnets, and linen dresses of a dove grey colour.

**Standard Books of Reference.**

**"The Nursing Profession: How and Where to Train." 2s. net; post free, 2s. 4d.**
**"The Nurses' Dictionary of Medical Terms." 2s.**
**"Burdett's Series of Nursing Text-Books." 1s. each.**
**"A Handbook for Nurses." (Illustrated.) 5s.**
**"Nursing: Its Theory and Practice." New Edition. 3s. 6d.**
**"Helps in Sickness and to Health." Fifteenth Thousand.**
**"The Physiological Feeding of Infants." 1s.**
**"The Physiological Nursery Chart." 1s.; post free, 1s. 3d.**
**"Hospital Expenditure: The Commissariat." 2s. 6d.**
**All these are published by THE SCIENTIFIC PRESS, Ltd., and may be obtained through any bookseller or direct from the publishers, 28 and 29 Southampton Street, London, W.C.**

### Travel Notes.

BY OUR TRAVELLING CORRESPONDENT.

### LXVI.—A WEEK'S HOLIDAY AT EASTER.

I PURPOSE to make a break in the series of papers on Rome by giving you two or three short accounts of suitable places on the Continent in which to spend a week at Easter, or a fortnight if you are fortunate enough to have so much leisure. It is quite time to be arranging plans for that season, and if any of you want help as to expenses and place of domicile let me beg you to ask me in *good time*. Why put off making arrangements to the last moment? You answer, perhaps, that the holiday may never come off, and all your labour be in vain. That is not my view at all. Granted you never have the holiday; you will have had great pleasure in planning and contriving for it, and anticipation is always pleasant when it lies in this direction.

There are many places which from their proximity to England are suitable for a short holiday: Holland is one of them, either Rotterdam or Amsterdam making a good centre. Then the banks of the Seine are a happy meeting ground for pedestrians, cyclists, artists, and photographers. In THE MIRROR of March 25th, 1899, I gave a full account of Candebec, which is my idea of the best stopping place in that locality. In the issue for July 22nd, 1899, you will find an account of Konigswinter, on the Rhine, a place admirably adapted for a short restful stay: the journey is quick, and the boats and trains make excursions an easy matter.

### ROTTERDAM.

First of all the question of cost is generally considered very important. You may stay a whole week for £5 with comfort if you take your ticket by the Netherlands Company's boat, which comes to 17s. return. These boats only run three times a week, so that a little arrangement is necessary. The journey occupies 15 hours, and if your holiday is limited you may not be able to spend so long a time in the transit. The next cheapest way is *via* Harwich, second-class return £1 4s. You can always go second class on these boats: they are clean and well appointed.

### THE COINAGE.

This is not quite so much to our advantage as in France and Italy. The coins chiefly in use are florins and cents. A florin is 1s. 8½d. of our money, and 100 cents go to a florin. Hotels are dearer than in Belgium, but there are cheap and good ones to be found. English money can often be used in the hotels, and the Dutch, though not (on a slight acquaintance) so agreeable as some other Continental nations, are honest and true, and will not take advantage of your inexperience.

### THE ATTRACTIONS OF HOLLAND.

These lie chiefly in the old-world cities, with their mediæval domestic architecture and the quaint narrow waterways, fine canals, and vast shipping in the ports. It is a place more suited to those who love to study past history, and who enjoy artistic effects, than for lovers of scenery. There really is no scenery, at least none of beauty, in Holland, though there is much to charm the eye. Neither is it a good cycling country on the whole. But walking is good, and for those who are perhaps tired and overworked, and do not want to make great exertions, it is specially convenient on account of the wonderfully numerous trains, boats, ferries, &c., by means of which every spot of interest can be easily reached without fatigue.

### BOOKS TO BE READ BEFORE STARTING.

Chief in interest I should place Motley's "Rise of the Dutch Republic." It is a most fascinating book and reads like one long novel. We hold our breath as we hear of the defence of Haarlem, its final surrender, and the hideous massacre of its brave defenders by the Spaniards; and even more thrilling is the account of the twelve months' siege of Leyden; of the glorious answer of the Burgomaster, Van der Werff, when the unfortunate townsfolk, worn out by six months' starvation, prayed him to yield. "Food I have none, else would I give it you; but if my death can be of use to you tear me in pieces and devour me. I have solemnly sworn that I will never surrender myself or my fellow citizens to the cruel and perfidious Spaniards, and I will die rather than violate my oath!"

After a siege of a year the Prince of Orange determined to break down the dykes and, by flooding the country, to reach the devoted garrison by water. This was accomplished on October 3rd, 1574. Six thousand people perished of famine and disease in that terrible year—a fact recorded on the Stadhuis.

An interesting novel dealing with old Holland at the time of the birth of Erasmus is "The Cloister and the Hearth," by Charles Reade. It treats of the romantic circumstances attending his birth at Rotterdam and childhood at Gouda. For an insight into modern Dutch home life the interesting stories of Maarten Maartens may be studied with advantage.

### THE AREA TO BE VISITED.

From Rotterdam, if time permits, you can visit The Hague, Leyden, Utrecht, Gouda, and Delft. It is possible also to see Haarlem, but that properly belongs to the neighbourhood of Amsterdam, and it is a great mistake to try and see too much in a short time. Your eyes, brains, and legs are overtired, and you see nothing thoroughly. Next week I will tell you of the principal sights of Rotterdam and its surroundings.

### TRAVEL NOTES AND QUERIES.

RULES IN REGARD TO CORRESPONDENCE FOR THIS SECTION.—All questioners must use a pseudonym for publication, but the communication must also bear the writer's own name and address as well, which will be regarded as confidential. All such communications to be addressed "Travel Editor, 'Nursing Mirror,' 28 Southampton Street, Strand." No charge will be made for inserting and answering questions in the inquiry column, and all will be answered in rotation as space permits. If an answer by letter is required, a stamped and addressed envelope must be enclosed, together with 2s. 6d., which fee will be devoted to the objects of the "'Hospital' Convalescent Fund." Any inquiries reaching the office after Monday cannot be answered in "The Mirror" of the current week."

SCOTLAND FOR A MONTH (E. H.).—You do not give me a pseudonym, so I must use your initials. I fear you cannot reckon upon your expenses coming to less than 10s. per day, counting everything, and you will be a good manager to keep within this sum. Scotland is a deplorably expensive country to travel in, partly because in the most delightful spots there is but little competition, and also the difficulty of transporting provisions is often considerable. (2) Ballachulish is an ideal spot, but, alas! far from cheap. The most reasonable hotel there is MacMillan's Temperance Hotel, near the pier. It is not bracing at Ballachulish, but is soft and moist. Dinnet, in the neighbourhood of Aberdeen, is cheaper, but though beautiful it is not so fascinating as Ballachulish.

ORVIETO (Wanderer).—You will see it is on the direct line between Milan and Rome, and therefore it will be quite easy to break your journey there. It is well worth seeing; the cathedral is magnificent and the position of the town most striking. Two days will show you everything, and I should advise the Albergo Tordi and Aquila Bianca. Your expenses need not exceed 8 lire per day.

THE LAKE OF LUCERNE IN SPRING (Robin).—You will think it beautiful. To my mind June is the month for Switzerland. I think you would find Brunnen a nice position and cheaper than Lucerne. Hôtel Pension Hirsch would suit you. The view from Brunnen is lovely and the walk on the Axenstrasse a thing to dream of. Your friend need not be fatigued with walking; the steamers are so frequent. By judicious use of these the whole of the lake can be explored even by the most humble pedestrian.

